# Atomically Precise Metal Nanoclusters for Near-Infrared-II Photonics

**DOI:** 10.1021/acs.accounts.5c00782

**Published:** 2026-01-13

**Authors:** Zhongyu Liu, Avirup Sardar, Sihan Chen, Yitong Wang, Rongchao Jin

**Affiliations:** Department of Chemistry, 6612 Carnegie Mellon University, Pittsburgh, Pennsylvania 15213, United States

## Abstract

Light in the near-infrared-II (NIR-II, 1000–2500 nm) region has enabled groundbreaking advances in photonic technologies, including long-distance optical communication, deep-tissue optical imaging, noninvasive neuromodulation, and high-efficiency solar energy conversion. Traditional NIR-II-responsive materials, such as rare-earth nanoparticles, carbon nanotubes, quantum dots, and organic chromophores, have achieved important progress. However, their performance is often constrained by intrinsic drawbacks, including narrow spectral response, low quantum yields, toxicity, and/or poor stability.

Recently, atomically precise metal nanoclusters (NCs), which bridge the gap between small molecules (e.g., complexes) and plasmonic nanoparticles, have emerged as a transformative platform for NIR-II photonics. Their tailorable compositions and atomic-level geometric structures give rise to versatile electronic structures, enabling highly controllable NIR-II absorption and emission and precise structure–property correlations. To date, metal NCs have demonstrated superior sensitivity in NIR-II light absorption, broad spectral responsiveness, and high photon-generation efficiency, outperforming many conventional NIR-II materials. These attributes make metal NCs particularly attractive for applications requiring high optical performance, spectral tunability, and biocompatibility.

In this Account, we summarize recent progress in the design, synthesis, and functionalization of NIR-II-responsive metal NCs. We highlight three major design principles that have driven advances in this field: (1) structural anisotropy, which promotes electron delocalization and enhances radiative transitions; (2) heteroatom doping, which modifies electronic transition dipoles and exciton relaxation pathways; (3) ligand engineering, which modulates energy dissipation within NCs and between NCs and their surrounding environment. Together, these approaches offer a versatile framework for controlling NIR-II photon absorption, conversion, and emission at the atomic scale.

Additionally, we discuss emerging applications of NIR-II-active metal NCs in deep-tissue optical bioimaging, photothermal therapy, and photocatalysis. The integration of precise structural control with tunable NIR-II optical properties opens new frontiers for next-generation photonic systems, where light manipulation at the atomic level can translate into transformative advances in biomedicine, sensing, and renewable energy technologies. Looking forward, continued exploration of novel NC structures, dopant chemistry, and surface functionalization will further expand the potential of metal NCs in NIR-II photonics, bridging the gap between fundamental discoveries and real-world applications.

## Graphical abstract

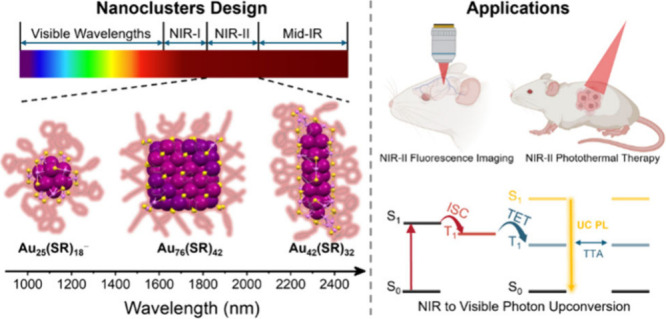

## Key References

-

Liu, Z. ; Zhou, M. ; Luo, L. ; Wang, Y. ; Kahng, E. ; Jin, R. , Elucidating the Near-Infrared Photoluminescence Mechanism of Homometal and Doped M_25_(SR)_18_ Nanoclusters. J. Am. Chem. Soc. 2023, 145, 19969–19981.37642696 10.1021/jacs.3c06543PMC10510323 ^1^
-

Liu, Z. ; Hu, X. ; Luo, L. ; He, G. ; Mazumder, A. ; Gunay, E. ; Wang, Y. ; Dickey, E. C. ; Peteanu, L. A. ; Matyjaszewski, K. , Near-Infrared to Visible Photon Upconversion with Gold Quantum Rods and Aqueous Photo-Driven Polymerization. J. Am. Chem. Soc. 2025, 147, 28241–28250.40717289 10.1021/jacs.5c08826PMC12333327 ^2^
-

Luo, L. ; Liu, Z. ; Kong, J. ; Gianopoulos, C. G. ; Coburn, I. ; Kirschbaum, K. ; Zhou, M. ; Jin, R. , Three-Atom-Wide Gold Quantum Rods with Periodic Elongation and Strongly Polarized Excitons. Proc. Natl. Acad. Sci. U.S.A. 2024, 121, e2318537121.38412123 10.1073/pnas.2318537121PMC10927531 ^3^
-

Wang, Y. ; Liu, Z. ; Mazumder, A. ; Gianopoulos, C. G. ; Kirschbaum, K. ; Peteanu, L. A. ; Jin, R. , Tailoring Carbon Tails of Ligands on Au_52_(SR)_32_ Nanoclusters Enhances the Near-Infrared Photoluminescence Quantum Yield from 3.8 to 18.3%. J. Am. Chem. Soc. 2023, 145, 26328–26338.37982713 10.1021/jacs.3c09846PMC10704554 ^4^

## 1 Introduction

The second near-infrared (NIR-II) window, also referred to as the short-wave infrared (SWIR) range, spans the wavelength from ∼1000 to 2500 nm in the electromagnetic spectrum. Light in this region experiences reduced Rayleigh scattering due to its longer wavelength than visible light and lower absorption by many natural and engineered materials, enabling deeper penetration into/through complex media. These optical properties have enabled transformative advances in photonic technologies that are challenging or impossible at shorter wavelengths, including optical communication,^5^ deep-tissue optical imaging,^6,7^ noninvasive neuromodulation,^8,9^ and photothermal cancer therapies.^10^ Additionally, NIR-II light accounts for a substantial fraction of the solar spectrum (∼20–25%). Yet, conventional silicon-based photovoltaic devices are unable to efficiently harvest these photons because the silicon bandgap is too large to absorb the low-energy SWIR photons.^11^ Therefore, developing optical materials that can capture, convert, and generate SWIR photons is crucial for enhancing solar-energy conversion, extending the reach of biomedical imaging and therapy, as well as achieving other next-generation photonic technologies.

Although research on NIR-II-responsive materials dates back to the late 20th century, creating systems that are both highly efficient and precisely controllable remains a major challenge. Well-studied materials, such as rare-earth nanoparticles,^12^ carbon nanotubes,^13^ semiconductor quantum dots,^14^ and organic chromophores,^15,16^ have made significant progress, but each has inherent limitations. Rare-earth nanoparticles respond only to narrow and limited wavelength ranges; single-walled carbon nanotubes exhibit low quantum yields^17^ semiconductor quantum dots are constrained by a limited selection of elements, many of which are toxic,^18^ and these inorganic nanomaterials cannot be engineered with atomic-level precision. Organic chromophores, by contrast, can be precisely designed at the atomic scale, but they often suffer from low absorption cross sections, poor quantum efficiencies, limited photostability, and environmental sensitivity.^16^

Atomically precise metal nanoclusters (NCs) have recently attracted growing interest as a new class of NIR-II-responsive materials due to their extraordinary optical properties and atomic-level tunability.^19,20^ They can absorb and generate photons across the entire NIR-II window (Scheme 1), making them among the broadest NIR-II-responsive materials ever reported. The peak absorption coefficient (ε) can reach 10^6^ M^–1^cm^–1^,^3^ with the emission brightness (ε×Φ, where Φ is quantum efficiency) reaching 10^4^–10^5^ M^–1^cm^–1^,^21^ being orders of magnitude higher than that of conventional materials. Furthermore, NCs can be rendered water-soluble either by employing hydrophilic ligands or by encapsulation and wrapping strategies while maintaining excellent biocompatibility and low toxicity. Most importantly, their atomically precise structures^19^ enable direct correlation between the structure and optical properties, allowing for single-atom-level modifications—an unprecedented capability for inorganic nanomaterials.

**Scheme 1:**
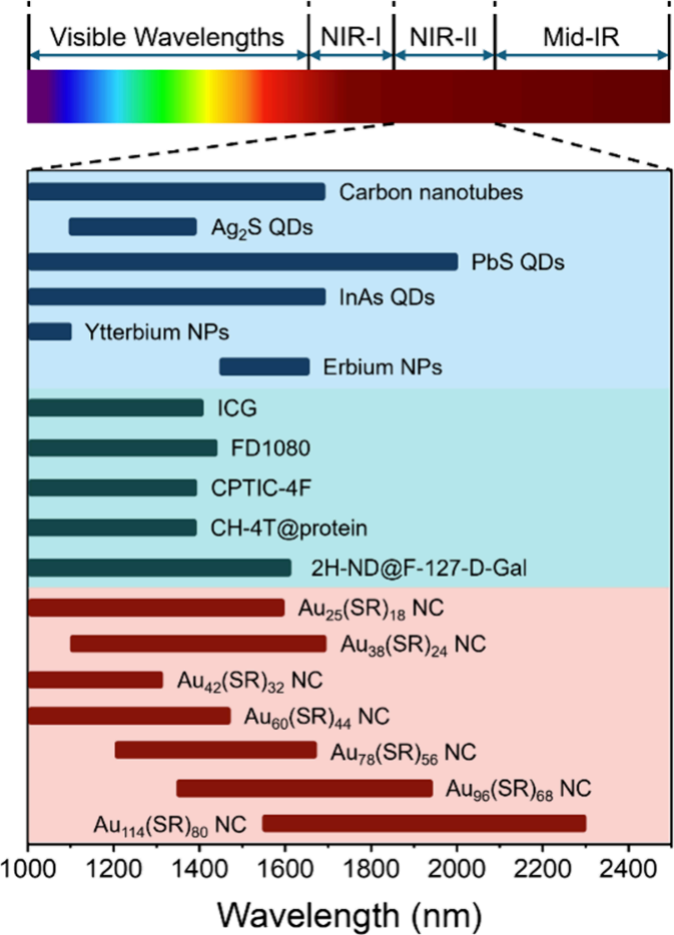
Emission Spectral Ranges of Different NIR-II Materials

Building on these advantages, continued efforts in understanding and optimization are needed to fully unlock the potential of metal NCs for next-generation photonic technologies. The electron dynamics of many NCs reflects the interplay of complex core–shell electronic structures, metal–ligand interactions, and spin–orbit coupling. In addition, the NIR-II photoluminescence (PL) quantum yields of many well-studied NCs remain modest (typically <1%), indicating significant room for enhancement through precise structural and electronic modulations.^22^ In this Account, we focus on the fundamental principles for tuning the optical properties of metal NCs in the NIR-II region and on establishing correlations between their optical responses and atomic structures. We highlight strategies such as stacking structural building blocks, heteroatom doping, and ligand engineering to enhance the capability of NCs to capture and emit NIR-II photons. Building on such insights, we discuss the rational design of NIR-II photonic systems with potential applications in biomedicine and photocatalysis, emphasizing how atomically precise engineering enables fine control over optical responses for high-performance applications.

## 2 Design of NIR-II Responsive Metal Nanoclusters

The structure of metal NCs comprises three hierarchical levels: metal core, staple motifs, and carbon tails of the protecting ligands.^23^ Modifications to any of them can alter the fundamental optical properties, including the absorption wavelength, absorption coefficient, emission wavelength, and quantum yield.^24^ This section outlines some design principles for engineering metal NCs with tailored NIR-II optical properties.

### 2.1 Tuning the Aspect Ratio of NCs

In organic molecules, it is well established that extending the π-conjugation system reduces the HOMO–LUMO gap, thereby red-shifting absorption and emission. Structural units such as carbon–carbon double bonds, carbon–carbon triple bonds, benzene rings, and thiophene rings are widely employed as building blocks to achieve narrow energy gaps in organic materials. A similar design principle applies to metal NCs, where strong NIR-II absorption and emission can be realized by engineering the metal core. Common structural units in the core of NCs, such as M_3_, M_4_, and M_13_ units (where, M = metal),^3,25,26^ serve roles analogous to π bonds in organic systems by acting as valence-electron-holding entities. When these units are directionally assembled into elongated architectures, the system behaves as quasi-one-dimensional: electrons are confined in the transverse directions but delocalize along the growth axis. This anisotropy simultaneously red-shifts the optical response and strengthens the transition dipole moment along the elongation axis, often yielding larger oscillator strengths than in isotropic NCs with comparable energy gaps.

The icosahedral M_13_ structure is among the most widely observed building blocks in metal NCs. A representative example is the classic Au_25_(PET)_18_^–^ NC (abbreviated *I*_*h*_-Au_25_, PET = phenylethanethiolate), which consists of an Au_13_ icosahedral core and six Au_2_(SR)_3_ staple motifs.^27^ The HOMO–LUMO transition of *I*_*h*_-Au_25_ gives rise to a dominant absorption peak at 675 nm and a split side peak at 784 nm (Figure 1A). Its emission spectrum features a peak at 1080 nm with 1% quantum yield (QY) and a Stokes shift of 0.4 eV.^28^ Fusion of two Au_13_ icosahedra gives rise to the biicosahedral Au_38_(SR)_24_,^29^ which presents a substantial red-shift of HOMO–LUMO transition to 1014 nm.^30^ Although the structure of Au_38_(PET)_24_ was solved more than a decade ago, its NIR-II emission profile was only recently determined using a DMBT-protected counterpart (DMBT = 2,4-dimethylbenzenethiolate).^31^ The emission centers at 1322 nm and extends beyond 1600 nm, suggesting that a stack of Au_13_ units via Au_3_-face-fusion can efficiently tune the response range of NCs for both capturing and generating NIR-II photons.

**Figure 1:**
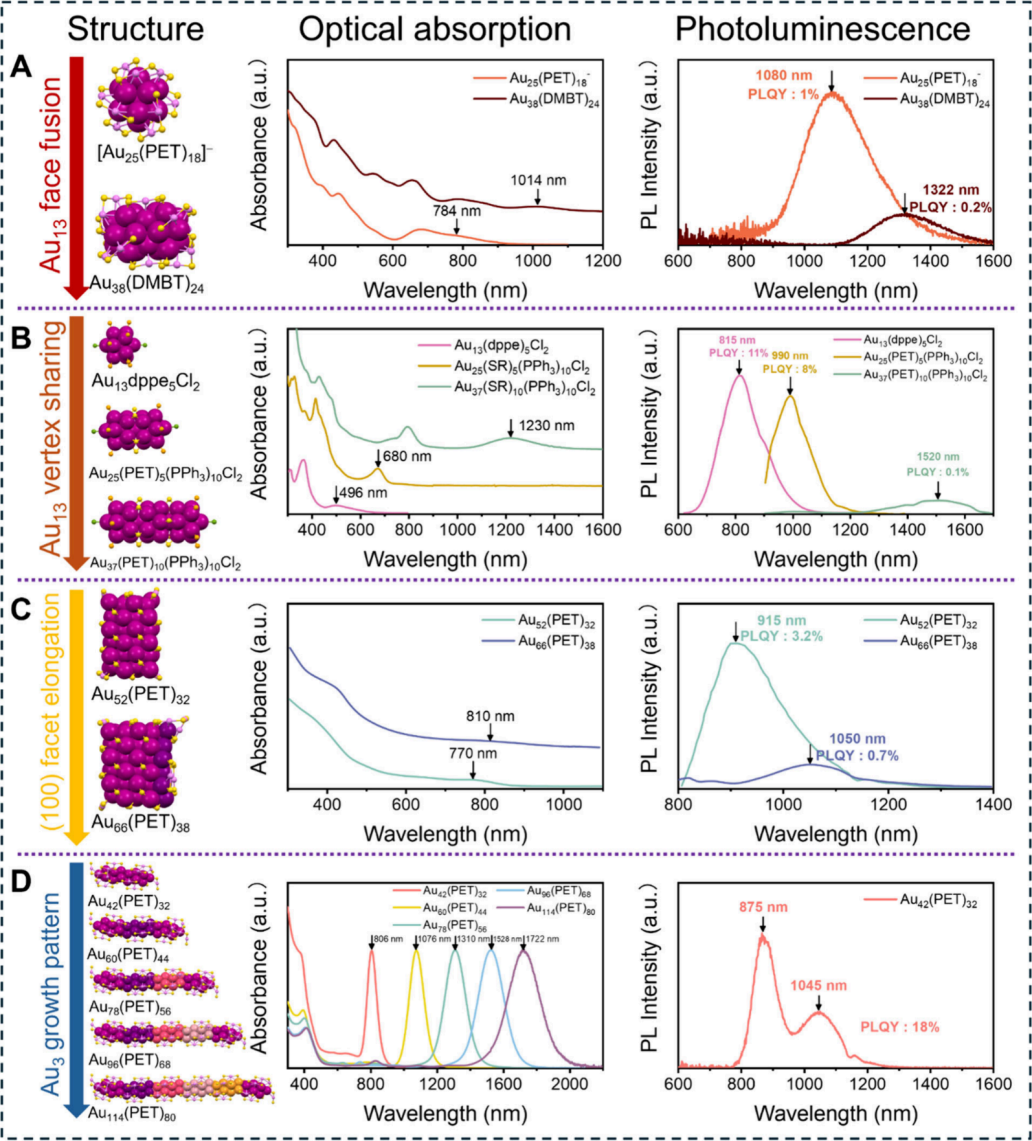
Structural, optical absorption, and PL evolution from (A) *I*_*h*_-Au_25_ to Au_38_ (Reproduced with permission from ref 31. Copyright 2025 American Chemical Society), (B) Au_13_ to rod-like Au_25_ and Au_37_ (Reproduced with permission from ref 35. Copyright 2021 Wiley-VCH), (C) Au_52_ to Au_66_ (Reproduced with permission from ref 39. Copyright 2024 Wiley-VCH), and (D) Au_42_ to Au_60_, Au_78,_ Au_96,_ and Au_114_ (Reproduced with permission from ref 3. Copyright 2024 National Academy of Science). Color code: magenta/pink/violet/reddish-pink/rose-pink/orange-yellow = Au, light orange = P, yellow = S, green = Cl, all R groups are omitted for clarity.

In addition to Au_3_-face-fusion, a linear assembly of *I*_*h*_-Au_13_ units through vertex sharing (Figure 1B) can start with [Au_13_(dppe)_5_Cl_2_]^3+^ (dppe = 1,2-bis­(diphenylphosphino)­ethane, abbreviated as Au_13_)^32,33^ and gives rise to [Au_25_(PET)_5_(PPh_3_)_10_Cl_2_]^2+^ (rod-Au_25_),^34^ and [Au_37_(PET)_10_(PPh_3_)_10_Cl_2_]^+^ (Au_37_).^26^ This structural evolution is accompanied by remarkable, progressive optical changes, with the HOMO–LUMO absorption peak red-shifting from 496 nm (Au_13_) to 680 nm (rod-Au_25_) and then to 1230 nm (Au_37_). Correspondingly, their emission maxima shift from 815 to 990 to 1520 nm, while the PLQY decreases from 11% to 8% to 0.1%.^35,36^ Linear growth based on *I*_*h*_-Ag_13_ and *I*_*h*_-Pt_1_@Ag_12_ units has also been reported,^37,38^ where the lowest-energy absorption band similarly redshifts with increasing aspect ratio; however, their potential for NIR-II photon generation remains unexplored. Beyond the *I*_*h*_-Au_13_ unit, elongation along the (100) facet (i.e., tetrahedron growth pattern) in Au_52_(PET)_32_ and Au_66_(PET)_38_ (Figure 1C) causes a reduction in energy gap and redshifts emission to NIR-II.^39^

Although the above examples extend NC emission into the NIR-II, PLQY decreases and the HOMO–LUMO transition’s oscillator strength weakens as the responsive wavelengths extend, limiting the NIR-II photon capture and generation efficiency. Remarkably, a recent discovery of the gold quantum rod series (Figure 1D), including Au_42_(PET)_32_, Au_60_(PET)_44_, Au_78_(PET)_56_, Au_96_(PET)_68_, and Au_114_(PET)_80_ (hereafter abbreviated as Au_42_, Au_60_, Au_78_, Au_96_, and Au_114_), has eliminated this intrinsic limitation.^3^ The Au_18_(PET)_12_ elongation-unit in this series comprises four Au_3_ layers for the core (i.e., octahedron growth pattern) and six Au­(PET)_2_ motifs on the surface. Unlike other rod-like NCs, these quantum rods exhibit the lowest-energy absorption band that is significantly stronger than their higher-energy features. The absorption coefficient increases progressively from Au_42_ to Au_96_ but somewhat decreases for Au_114_. Density functional theory (DFT) simulations reveal that the ultrasmall diameter of these rods promotes a high spatial overlap between the ground and excited-state electron densities, which intensifies as the rod length increases. The measured absorption coefficients range from 10^5^ to 10^6^ M^–1^ cm^–1^, demonstrating an exceptional NIR-II photon capture capacity that is rarely achieved in other materials.

The Au_42_ NC exhibits dual emission centered at 875 and 1045 nm, with a combined PLQY of 18% at 806 nm excitation (11.9% if 380 nm excitation).^21,40^ The emission spectra of Au_60_, Au_78_, Au_96_, and Au_114_ cover 1000–2300 nm, and some exhibit PLQYs exceeding 20% when excited at their HOMO–LUMO absorption peaks (detailed data to be published separately). The unprecedented NIR-II photon capture and emission capabilities of these quantum rods make them outstanding candidates for deep-tissue imaging, photothermal therapy, and NIR-II light-emitting diodes.

### 2.2 Doping Effect

A key feature that distinguishes metal NCs from other nanomaterials is their capacity for atomically precise single-atom modification, with heteroatom doping being one of the most effective strategies. Remarkably, even a single-atom change can dramatically alter a NC’s optical properties while preserving its original geometric framework, offering a highly controlled approach to achieving versatile optical responses across the electromagnetic spectrum.^41^

The *I*_*h*_-M_25_ (M = Au and Ag, Figure 2A,B) system provides a representative framework for gaining fundamental insights into the effect of heteroatom doping. Both *I*_*h*_-Au_25_ and *I*_*h*_-Ag_25_ possess a M@M_12_ icosahedral core protected by six M_2_(SR)_3_ staple motifs, forming a closed-shell eight-electron superatom configuration. When Cd and Hg atoms are introduced into the *I*_*h*_-Au_25_,^42^ they substitute a gold atom on the Au_12_ icosahedral shell (Figure 2A), with minimal change to the overall structural framework.^43^ However, the electronic and optical properties of *I*_*h*_-Au_25_ vary a lot with doping. The characteristic absorption peak of *I*_*h*_-Au_25_ at 678 nm shifts to 657 nm in CdAu_24_ and to 705 nm in HgAu_24_, while the 784 nm shoulder shifts to 755 nm in CdAu_24_ and disappears in HgAu_24_.^44,45^ Similarly, the emission maximum shifts from 1080 nm (*I*_*h*_-Au_25_) to 1045 nm (CdAu_24_) and 1170 nm (HgAu_24_), accompanied by changes in the PLQY from 1% to 1.7% and 0.3%, respectively. Temperature-dependent PL measurements (Figure 2C) further reveal that heteroatom doping not only modifies the electronic structure of *I*_*h*_-Au_25_ but also significantly alters the coupling between vibrational modes and electronic transitions (Figure 2D), leading to pronounced changes in electron dynamics and NIR-II photon generation efficiency.^1^

**Figure 2:**
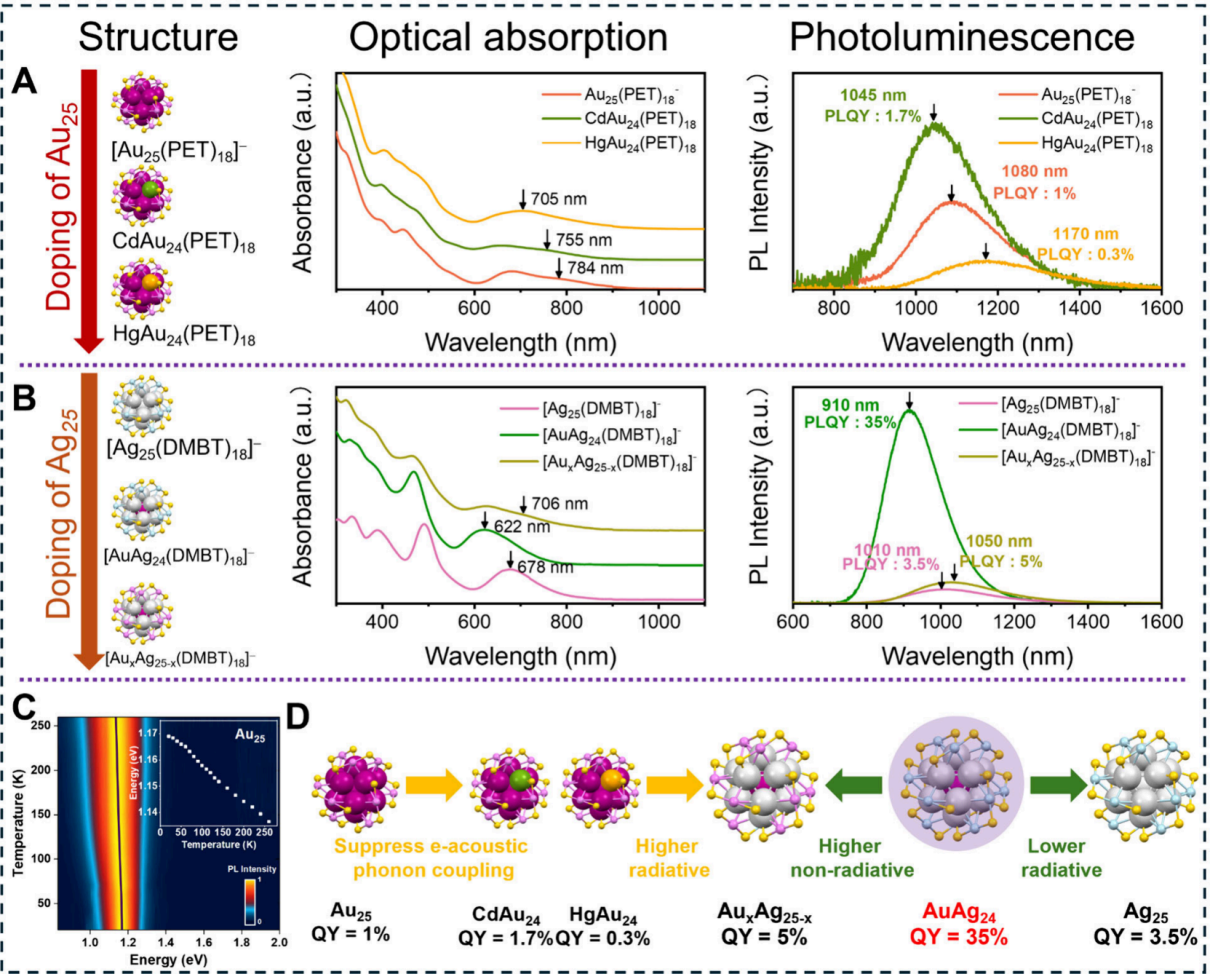
Structural, optical absorption, and PL evolution from (A) *I*_*h*_-Au_25_ to CdAu_24_ and HgAu_24_, (B) Ag_25_ to AuAg_24_ and Au_*x*_Ag_25‑*x*_. (C) Temperature-dependent PL spectra of *I*_*h*_-Au_25_. (D) Schematic illustration of the mechanism underlying the PLQY evolution among six NCs. Color code: magenta/pink = Au, silver/blue = Ag, green = Cd, Orange = Hg, yellow = S, and R groups are omitted for clarity. Reproduced with permission from ref 1. Copyright 2023 American Chemical Society.

In contrast, *I*_*h*_-Ag_25_, despite having the same geometric structure of *I*_*h*_-Au_25_, exhibits a markedly different electronic structure, displaying a single broad HOMO–LUMO transition at 678 nm rather than a split feature. Its emission is centered at 1010 nm, with a PLQY of 3.5% (Figure 2B). Introducing a gold atom into Ag_25_ replaces the central silver atom in the icosahedral core, forming an Au@Ag_12_ core and yielding an AuAg_24_ NC. This modification results in a blueshift of the absorption peak to 622 nm and the emission peak to 910 nm, accompanied by a 10-fold increase in PLQY to 35%.^46^ However, further doping with more gold atoms preferentially replaces silver atoms in the staple motifs, leading to a 706 nm absorption shoulder and a decrease in PLQY to 5%. Time-resolved PL measurements indicate that the radiative relaxation rate increases significantly when a gold atom is in the center of Ag_25_, likely due to the difference in electronegativity between silver and gold, which enhances the transition dipole. Temperature-dependent PL measurements reveal that, with additional gold doping into AuAg_24_, the electron-vibration coupling turns from a weak regime to a strong regime, significantly increasing nonradiative decay, hence, reducing PLQY (Figure 2D). Additionally, a transition from thermally activated delayed fluorescence to phosphorescence is observed as the number of doped gold atoms increases, suggesting that heteroatom doping can alter the energy dissipation pathway.^1^

In addition to the *I*_h_-M_25_ system, similar behavior was observed in the representative Ag_29_ NC.^24,41^ Ag_29_ consists of an *I*_h_-Ag_13_ core capped by an outer shell formed by the remaining 16 Ag atoms. Introducing a single Au dopant at the center of the *I*_h_-Ag_13_ core leads to a pronounced enhancement in emission intensity. Unlike the Ag_25_ doping, further Au incorporation into Ag_29_ results in a continuous increase in PLQY, which can be attributed to the different structure of the Ag_29_ outer shell.^41^

Overall, heteroatom doping is an efficient and versatile strategy for tuning the optical properties of NCs. It not only modifies the electronic states of NCs, thereby adjusting their electron dynamics and photon-capturing capabilities, but also influences the interaction between electronic and vibrational states, thereby controlling their efficiency in converting the absorbed photons into thermal energy or longer-wavelength photons.

### 2.3 Ligand Engineering

The protecting ligands act like the “skin” of NCs, stabilizing them as discrete entities, controlling their interactions with the surrounding environment^22^ and modulating their electronic states through metal-to-ligand or ligand-to-metal charge transfer. Ligand engineering (Figure 3A,B) plays a critical role in modulating photoexcitation energy dissipation pathways.

**Figure 3:**
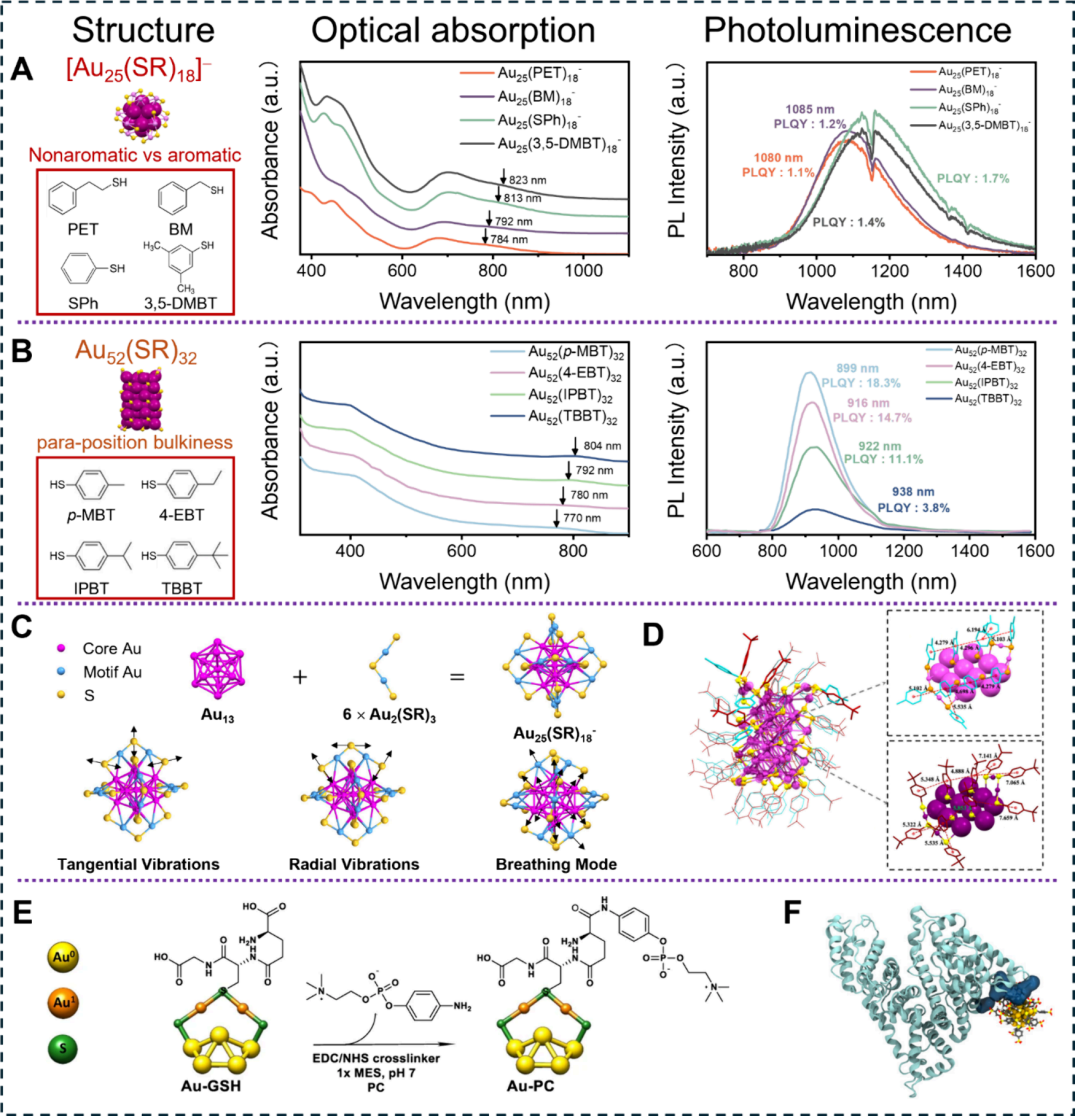
Structural, optical absorption, and PL evolution from (A) Au_25_(PET)_18_ to Au_25_(BM)_18_, Au_25_(SPh)_18_, and Au_25_(3,5-DMBT)_18_. Reproduced with permission from ref 28. Copyright 2022 American Chemical Society. (B) Au_52_(*p*-MBT)_32_ to Au_52_(4-EBT)_32_, Au_52_(IPBT)_32_, and Au_52_(TBBT)_32_. Color code: magenta/pink = Au, yellow = S, and R groups are omitted for clarity. Reproduced with permission from ref 4. Copyright 2023 American Chemical Society. (C) Structure and vibrational modes of *I*_*h*_-Au_25_. (D) Overlay of Au_52_(*p*-MBT)_32_ and Au_52_(TBBT)_32_ SCXRD structures. Color code: purple/magenta = gold; yellow/orange = sulfur; red = carbon on TBBT; light blue = carbon on *p*-MBT. (E) Post functionalization of the Au-SG NC. Reproduced with permission from ref 47. Copyright 2022 Springer Nature. (F) Computational modeling of the specific binding sites for Au_25_(*p*-MBS)_18_ on bovine serum albumin (*p*-MBS = para-mercaptobenzenesulfonic acid). Reproduced with permission from ref 48. Copyright 2025 American Chemical Society.

Liu et al. adopted *I*_*h*_-Au_25_ (Figure 3A) as a representative model for exploring how the protecting ligands affect excited-state relaxation dynamics and energy dissipation behavior.^28^ When the protecting ligand is changed from PET to benzylmercaptan (BM), thiophenolate (SPh), and 3,5-dimethylbenzenethiolate (3,5-DMBT), the lowest-energy absorption band exhibits a slight redshift, due to stronger electron delocalization in aromatic ligand-protected NCs. Although the ligands make only a minor contribution to the HOMO–LUMO transition, temperature-dependent optical absorption measurements reveal that they profoundly affect the relaxation pathway associated with the HOMO–LUMO transition. The high-frequency Au–S vibrational modes (Figure 3C) within the Au_2_(SR)_3_ staple motifs are dominant in mediating the electron relaxation in aliphatic thiolate-protected NCs such as Au_25_(PET)_18_ and Au_25_(BM)_18_.^49^ In contrast, the coupling between the Au–S vibrational modes and the HOMO–LUMO electronic transition is significantly suppressed in aromatic thiolate-protected NCs, including Au_25_(SPh)_18_ and Au_25_(3,5-DMBT)_18_, leading to suppressed nonradiative relaxation. Consistent with this interpretation, differences in emission intensity are observed in both solution and solid-state films.

Additionally, Wang et al. systematically investigated the effect of *para*-substituents on aromatic ligands in Au_52_(SR)_32_ NCs (Figure 3B).^4^ Au_52_ protected by *p-*methylbenzenethiolate (*p*-MBT, R = – C_6_H_4_–CH_3_), *p*-ethylbenzenethiolate (4-EBT, R = – C_6_H_4_–CH_2_CH_3_), *p*-isopropylbenzenethiolate (IPBT, R = – C_6_H_4_–CH­(CH_3_)_2_), and *p*-*tert*-butylbenzenethiolate (TBBT, R = – C_6_H_4_-*t*Bu) were synthesized and compared. As the number of methyl groups on the para substituent increases, the lowest-energy absorption peak of Au_52_ exhibits a slight redshift from 770 to 804 nm, with the overall absorption profiles remaining nearly identical. A similar trend is observed for the emission peaks of the four NCs, reflecting a modest increase in the electron-donating ability of the substituents. However, the PLQY varies dramatically, with Au_52_ capped by the least bulky (*p*-MBT) ligand showing the highest PLQY (18.3%), and the one protected by the bulkiest (TBBT) ligand exhibits only 3.8%. Single-crystal X-ray diffraction (SCXRD) analysis revealed that the number and steric bulkiness of methyl groups on the *para*-position significantly modified the surface packing of ligands, as the *p*-MBT-protected NC exhibits the strongest intermolecular π–π interactions between ligands (Figure 3D). Moreover, temperature-dependent optical absorption measurements indicated that nonradiative decay via multiphonon relaxation pathways mediated by C–H vibrational modes is substantially suppressed in Au_52_(*p*-MBT)_32_.

Beyond the complete ligand exchange, surface functionalization through chemical modification of existing ligands enables coupling between metal NCs and small molecules or biomacromolecules. For instance, Baghdasaryan et al. (Figure 3E) covalently linked glutathione-protected Au_25_(SG)_18_ (SG = glutathione) to 4-aminophenylphosphorylcholine via amide bond formation, which enhanced emission efficiency and improved the signal-to-noise ratio in NIR-II fluorescence imaging.^47^ Moreover, Zhang et al. (Figure 3F) bound Au_25_(*p*-MBS)_18_ NCs to bovine serum albumin (BSA) through ionic interactions, enabling electron transfer from BSA to the NCs and dramatically enhancing NIR-II emission.^48^

Ligand engineering modulates the optical properties of metal NCs through two mechanisms. First, the ligands affect the transfer and delocalization of the valence electrons in metal NCs, thereby tuning their electronic structure, charge-transfer characteristics, and optical absorption behavior.^24^ Concurrently, the ligands govern how the excited-state electrons of NCs dissipate their energy. Generally, rigid, π-conjugated ligands tend to suppress vibrational coupling and favor radiative relaxation, thereby enhancing PLQY.^28^ In contrast, flexible or aliphatic ligands facilitate nonradiative relaxation and promote more effective photothermal conversion.

### 2.4 Quantum Platelet

Very recently, Wang et al. reported a breakthrough in the synthesis of highly luminescent two-dimensionally anisotropic metal nanoclusters. Under elevated-temperature conditions in a size-focusing process with excess thiol, an Au_76_(*p*-MBT)_42_ quantum platelet was obtained. The thermally activated growth enables side-facet (010) extension of the Au_52_(*p*-MBT)_32_ nanorod, yielding a structure with a square face-centered cubic core of 1 nm edge length (Figure 4A).^50^ The optical absorption spectrum of Au_76_ shows a strong HOMO–LUMO transition at 810 nm and less distinct bands at 370 and 470 nm (Figure 4B). In toluene solution, Au_76_ exhibits bright 970 nm emission with a PLQY of 30%, which increases to 40% under deaerated conditions. To probe its excited-state dynamics, femtosecond (fs) and nanosecond (ns) transient absorption (TA) measurements were performed using a 400 nm pump. Within the first picosecond (ps), broad excited-state absorption (ESA) appears at 550 and 900 nm, accompanied by ground-state bleaching (GSB) from 700–850 nm, consistent with the steady-state absorption (Figure 4C). Over time, the ESA narrows at 550 nm while a broad NIR ESA rises as the initial excited-state decays. The ns-TA spectra (Figure 4D) reveal a long-lived (∼750 ns) triplet state. Kinetic traces at 900 nm (S_1_ absorption) and 1300 nm (T_1_ absorption) show a sequential S_1_ → T_1_ → S_0_ process (Figure 4E). Global fitting of the fs-TA data yields an ISC lifetime of 0.7 ps. The evolution-associated difference spectra (EADS) are shown in Figure 4F, in which the first species is attributed to the S_1_ state and the second one to the T_1_ state. Taken together, these structural and excited-state insights help explain the photophysical behavior of Au_76_, and the substantially stronger PL of Au_76_ (30%) compared with Au_52_ (18%) is attributed to its more compact core, which suppresses nonradiative decay.

**Figure 4:**
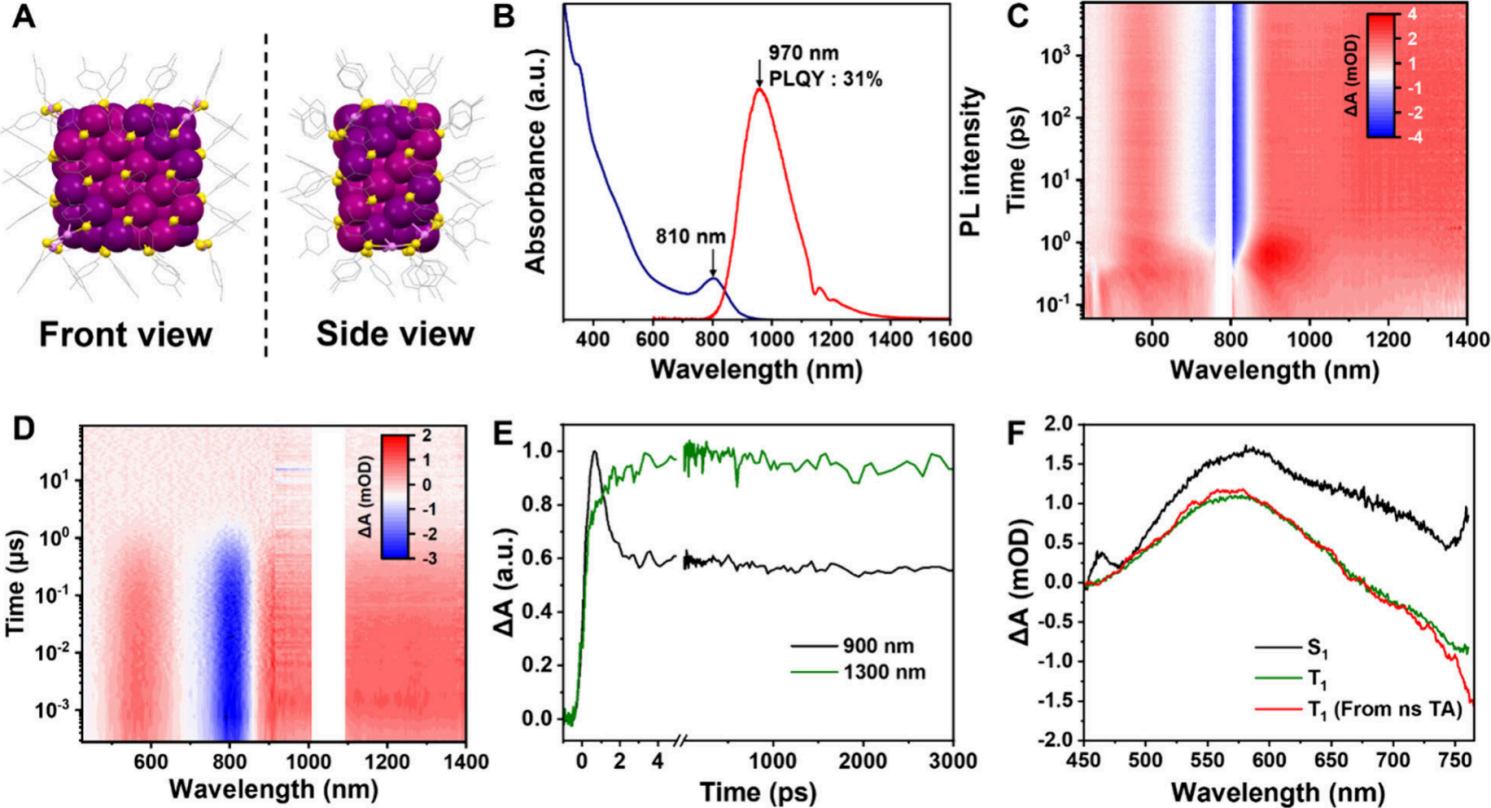
(A) Structure of Au_76_(*p*-MBT)_42_. Color code: purple/magenta/pink = Au, yellow = S. (B) Spectra of optical absorption (blue line), and emission (red line) of Au_76_ in solution. (C) fs-TA data map of Au_76_. (D) ns-TA data map of Au_76_. (E) Kinetics of S_1_ signal at 900 nm and T_1_ signal at 1300 nm. (F) Global analysis of fs-TA of Au_76_. Reproduced with permission from ref 50. Copyright 2025 American Chemical Society.

## 3 Applications for NIR-II Photonics

Atomically precise metal NCs exhibit extraordinary NIR-II optical properties and versatile tunability through compositional and surface engineering. These combined advantages have enabled diverse NIR-II photonic applications, including deep-tissue optical imaging, photothermal therapy, and upconversion photocatalysis.

### 3.1 Metal NCs in Aqueous Phase

Although SWIR light was initially employed for night vision, fiber-optic communication, and industrial inspection in the late 20th century, its exceptional penetration capability through scattering media has recently revealed great potential for deep-tissue optical imaging in biological systems. To harness NIR-II light for biomedical applications, materials must be both water-soluble and biocompatible. Thus, developing water-dispersible metal NCs or NC-based nanocomposites with NIR-II response is essential.

One important strategy to obtain water-soluble metal NCs is the use of hydrophilic protecting ligands during the synthesis. Early examples include glutathione, *p*-mercaptobenzoic acid, etc. (Figure 5A,B), which enabled the successful preparation of a range of gold NCs.^51–53^ Among them, Au_22_(SG)_18_ and Au_25_(SG)_18_ have been extensively studied for photonic and bioimaging applications.^47,54^ More recently, hydrophilic heterocyclic ligands have been introduced to enhance PLQY and promote crystallization of water-soluble NCs through stronger intermolecular interactions.^55,56^ Beyond small molecules, biomacromolecules such as DNA have also been employed as ligands to produce NIR-responsive silver NCs.^57^ Sullivan et al. further developed N-heterocyclic carbene (NHC)-protected gold NCs functionalized with triethylene glycol monomethyl ether groups, providing a robust route to transfer NCs into the aqueous phase while preserving their core structures.^58^ Despite near-unity PLQYs achieved for visible-emitting, water-soluble NCs, efficient photon generation in NIR-II remains challenging, as only Au_25_(SR)_18_ and Au_44_(SR)_26_ have demonstrated NIR-II emission, both with low QYs.^59^

**Figure 5:**
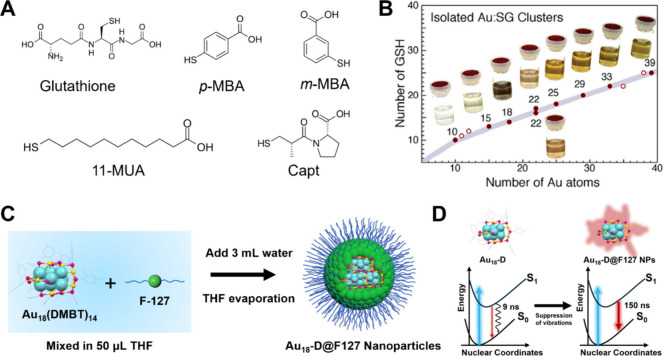
(A) Representative water-soluble thiols for the synthesis of gold NCs. (B) Plot showing the relationship between the number of Au atoms and −SG ligands in SG-protected gold NCs. Reproduced with permission from ref 52. Copyright 2005 American Chemical Society. (C) Schematic formation process of Au_18_-D@F127 NPs. (D) Schematic illustration of the excited-state dynamics of Au_18_-D and Au_18_-D@F127 NPs. Reproduced with permission from ref 31. Copyright 2025 American Chemical Society.

To address this limitation, postsynthetic phase transfer has emerged as an effective approach for transferring NIR-II–responsive NCs from organic media to water while preserving their structure and optical properties. Various encapsulation methods, including the use of cyclodextrins, proteins, metal–organic frameworks, and silica nanoparticles, have been explored.^60,61^ However, these approaches often suffer from drawbacks such as a limited encapsulation amount, increased optical scattering, and poor stability under physiological conditions. To address these issues, Liu et al. recently introduced an amphiphilic polymer (F127) wrapping strategy that enables efficient transfer of organically soluble NCs into aqueous phase without altering their structure or optical properties (Figure 5C,D).^31^ The resulting Au_18_@F127 nanoparticles, still below 2 nm in size, exhibit negligible scattering, excellent stability in simulated biofluids, and a 10-fold enhancement in PL intensity due to suppressed nonradiative relaxation via intercluster interactions. Importantly, this versatile strategy can be extended to other NCs, enabling water-phase NIR-II photon generation in Au_38_(SR)_24_ and Au_42_(SR)_32_ NCs.

Overall, these developments provide a framework for integrating the extraordinary NIR-II optical responses of metal NCs into aqueous environments, thereby expanding their applications in biomedical imaging, phototherapy, and other photonic technologies.

### 3.2 NIR-II Optical Bioimaging and Photothermal Therapy

Owing to reduced light scattering, absorption, and autofluorescence by biological tissues, the NIR-II range for in vivo fluorescence imaging enables noninvasive optical imaging at millimeter-scale depths within live biological organisms. Among the diverse NIR-II imaging agents reported to date, gold NCs offer distinct advantages in biosafety and biocompatibility compared with inorganic nanomaterials such as carbon nanotubes and quantum dots. In addition, unlike FDA-approved organic dyes such as indocyanine green, which suffer from rapid hepatic uptake and short plasma half-lives (2–4 min), metal NCs allow tunable surface engineering to achieve prolonged blood circulation times (>0.5 h), enabling extended-duration deep-tissue imaging. Together, these characteristics make metal NCs promising probes for advancing NIR-II fluorescence imaging, facilitating deeper insights into fundamental biology, and supporting clinical diagnostics and therapeutic applications.

Water-soluble ligand-protected Au_25_(SR)_18_ NCs (e.g., glutathione) are among the most extensively studied NCs for NIR-II fluorescence imaging. Xie and Zhang’s groups systematically investigated the effects of various ligands on PLQY of water-soluble Au_25_ and enhanced emission intensity through heteroatom doping with Er, Ag, Cu, and Zn.^62^ The resulting bright probe exhibits strong NIR-II emission capable of efficiently penetrating the mouse skull, enabling high-contrast imaging of cerebral blood vessels. This high-quality NIR-II fluorescence imaging technique was further applied to real-time monitoring of cerebrovascular injury in ischemic stroke models via dynamic fluorescence imaging, as well as for in vivo visualization of tumor metastasis (Figure 6A).^62^ Later, Li et al. demonstrated the effectiveness of Au_25_(SG)_18_ as an NIR-II probe for high-resolution bone imaging, underscoring their potential for fluorescence-guided implant surgery (Figure 6B).^63^ Additionally, Ma et al. employed Au_22_(SG)_18_ to monitor cisplatin-induced renal injury within 20–120 min and visualize it in three dimensions via NIR-II light-sheet microscopy, demonstrating its potential for clinical diagnosis and monitoring of acute kidney injury (Figure 6C).^64^

**Figure 6:**
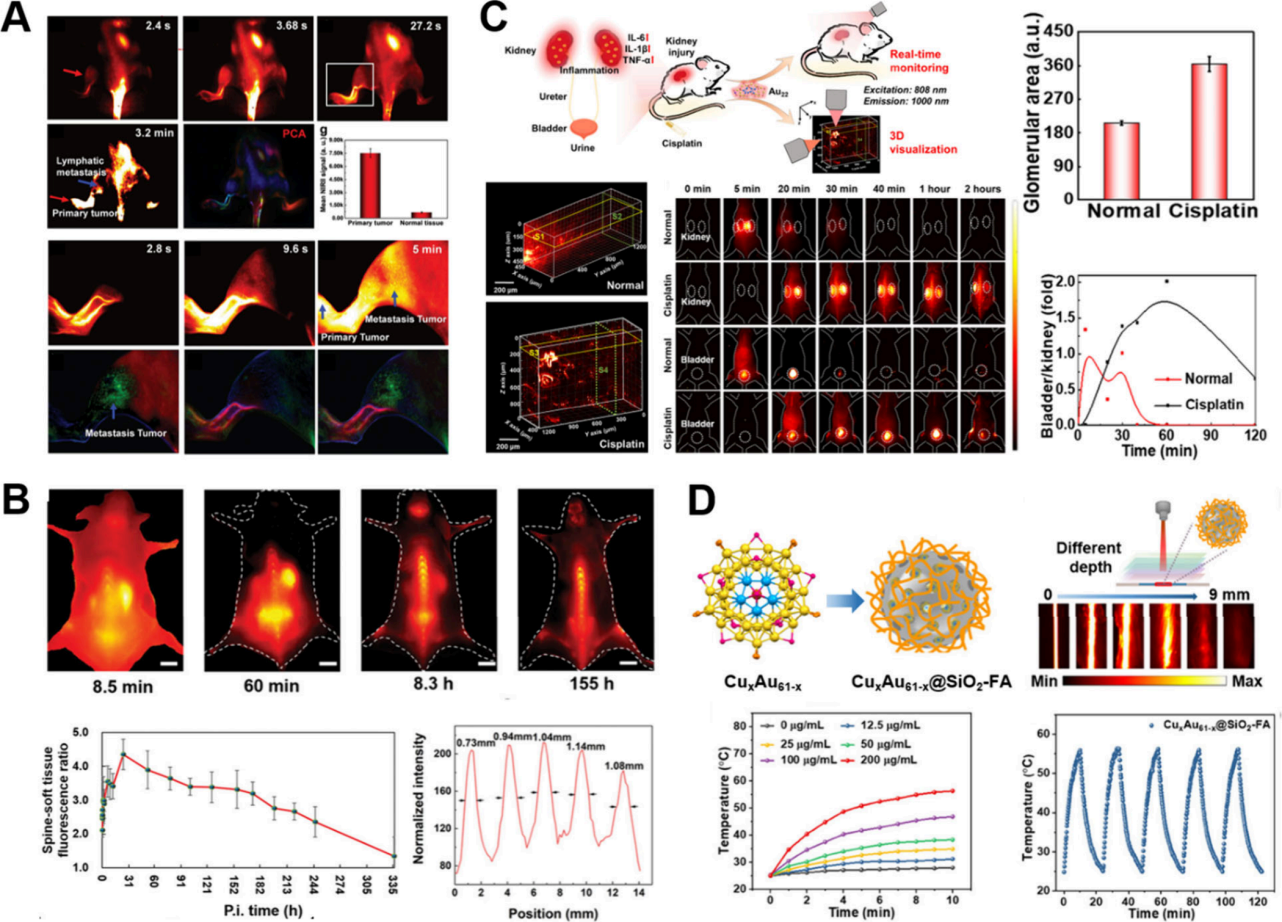
(A) Dynamic tumor metastasis imaging with Au NCs and PCA images in the NIR-II window from 1300 to 1700 nm under an excitation of 808 nm. Reproduced with permission from ref 62. Copyright 2019 Wiley-VCH. (B) High-resolution bone-targeted fluorescence image of C57BL/6 mice for the whole body in prone posture at various time courses. Reproduced with permission from ref 63. Copyright 2020 Wiley-VCH. (C) NIR-II imaging of light-sheet microscopy and the real-time monitoring process of Au NCs. Reproduced with permission from ref 64. Copyright 2023, American Association for the Advancement of Science. (D) Cu_*x*_Au_61‑*x*_ NCs for NIR-II imaging and photothermal therapy. Reproduced with permission from ref 66. Copyright 2025 Wiley-VCH.

In addition to fluorescence imaging, the strong tissue penetration of NIR-II light efficiently triggers the photothermal effect of NCs in deep tissues, enabling both photoacoustic imaging and photothermal therapy. Unlike plasmonic nanoparticles, NCs generate heat primarily through nonradiative decay via internal conversion and interactions between NCs and the environment, modulated by their size, ligands, and electronic structure.

In 2023, Li and colleagues encapsulated Au NCs around gold nanorods, achieving a temperature rise of 35.1 °C under 1064 nm laser irradiation (1 W/cm^2^).^65^ The designed nanocomposite acted as “thermal bombs”, allowing tumor localization via NIR-II photoacoustic imaging and precise tumor ablation through photothermal therapy. Xu et al. developed a multifunctional, water-dispersible Cu_*x*_Au_61–*x*_@SiO_2_–FA nanoplatform (Figure 6D) with excellent biocompatibility, efficient photothermal effect and strong NIR-II luminescence, achieving outstanding performance in NIR-II luminescence imaging-guided tumor phototherapy.^66^ In the future, the emerging Au quantum rods^3^ and quantum needles^67^ may further offer promising ways for next-generation NIR-II photothermal agents with enhanced optical and therapeutic performance.

### 3.3 Triplet–Triplet Annihilation Upconversion

Triplet–triplet annihilation upconversion (TTA-UC) converts two low-energy photons into one higher-energy photon, enabling breakthroughs in photocatalysis, photovoltaics, and neuromodulation.^8,11,68^ A typical TTA-UC system comprises a triplet sensitizer (S) and an emitter (E). Upon absorption of a low-energy photon, the ground-state sensitizer (^1^S) is excited and undergoes intersystem crossing to form an excited triplet state (^3^S*), which subsequently transfers its energy to the emitter via triplet energy transfer, generating a long-lived triplet excited state (^3^E*). When two ^3^E* species interact, triplet–triplet annihilation occurs, producing one emitter in the ground state (^1^E) and the other in a higher-energy singlet excited state (^1^E*), from which upconverted fluorescence is emitted.^69^ However, achieving efficient NIR-to-visible upconversion remains extremely challenging due to limited triplet populations, inefficient photosensitization, and rapid exciton decay across narrow energy-gaps.

Metal NCs offer a promising platform to overcome these barriers owing to their extraordinary and tunable optical responses in the NIR-II region. Recently, Liu et al. demonstrated upconversion of 1045 nm phosphorescence into 610 nm fluorescence using a system composed of Au_42_(SR)_32_ quantum rods as the sensitizer and 5,11-bis­(triethylsilylethynyl)­anthradithiophene (TES-ADT) as the annihilator (Figure 7A,B).^2^ Benefiting from the strong photosensitization of Au_42_, its long triplet lifetime, and efficient energy transfer to TES-ADT, the system achieved a 6.7% QY, 0.5 eV anti-Stokes shift, and low threshold intensity of 90 mW/cm^2^ (Figure 7C), outperforming most existing NIR-to-visible TTA-UC materials. To enable aqueous applications, upconversion nanodroplets were encapsulated within a silica shell (Figure 7D), yielding Au_42_/TES-ADT@SiO_2_ nanoparticles that emit at 560 nm (Figure 7E,F). The emitted light can drive photoinduced atom-transfer radical polymerization (photo-ATRP) and form hydrogels in water (Figure 7G). This system not only enables photopolymerization in aqueous phase with high efficiency but also holds broad potential for self-healing implants and minimally invasive therapies.

**Figure 7:**
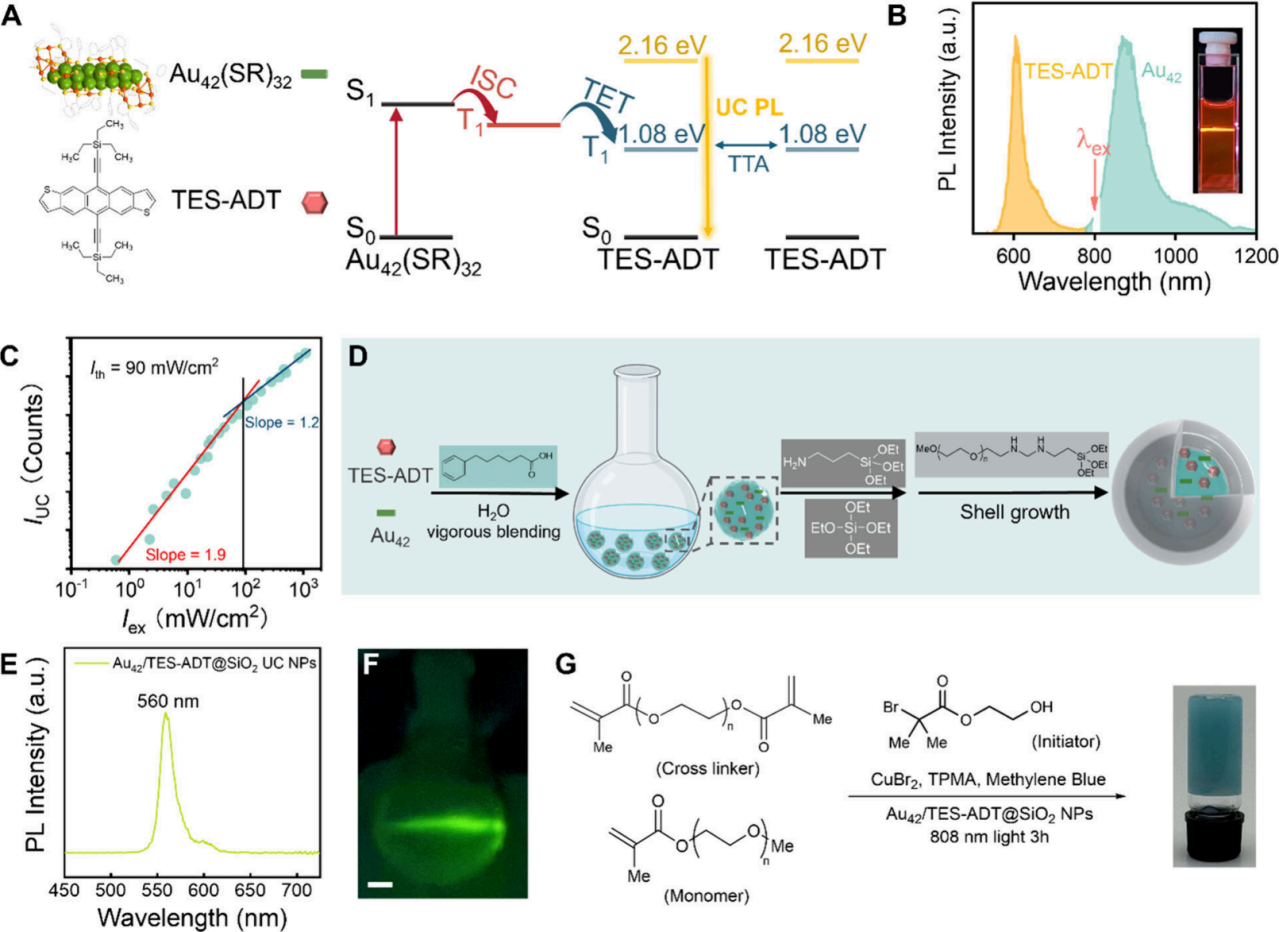
(A) Schematic illustration of NIR-to-visible photon upconversion through a combination of Au_42_(SR)_32_ and TES-ADT. (B) TTA-UC emission spectra under 808 nm continuous laser excitation (inset: photograph of NIR-to-yellow upconversion in a cuvette). (C) Dependence of the UC emission intensity on the incident laser fluence. (D) Overview of the synthesis of Au_42_/TES-ADT@SiO_2_ TTA-UC NPs. (E) Visible region emission spectrum of Au_42_/TES-ADT@SiO_2_ TTA-UC NPs. (F) Photograph of a suspension of Au_42_/TES-ADT@SiO_2_ NPs under 808 nm laser, scale bar: 1 cm. (G) Schematic illustration of Au_42_/TES-ADT@SiO_2_ NPs enabled photo-ATRP reaction to form a cross-linked hydrogel. Reproduced with permission from ref 2. Copyright 2025 American Chemical Society.

Nevertheless, NIR-II-excited TTA-UC has not yet been realized using metal NCs as sensitizers. Anisotropic NCs hold great promises for photosensitizing in the NIR-II region, but further engineering is needed to achieve long-lived (hundreds of nanoseconds to microsecond-scale) triplet states for efficient energy transfer from NCs to molecular acceptors.

## 4 Conclusions and Perspectives

In this Account, we have summarized recent progress in the design and applications of NIR-II-responsive, atomically precise metal NCs. Advances in anisotropic NC synthesis, heteroatom doping, and ligand engineering have provided powerful strategies to tune and enhance their optical responses in the NIR-II region. Meanwhile, significant efforts have been devoted to improving their water solubility, enabling the remarkable optical properties of metal NCs to be harnessed for applications in bioimaging, phototherapy, and photocatalysis.

Despite these achievements, several fundamental questions remain open and call for future efforts.

First, while anisotropic metal NCs often display enhanced optical performance, such as stronger absorption, higher PLQYs, and longer exciton lifetimes, the mechanisms governing their anisotropic growth are still not well understood.^3^ Systematic studies of their formation pathways will be crucial for the rational design of functional NCs.

Second, integrating rare-earth elements into metal NCs presents an exciting opportunity for NIR-II light generation. While rare-earth dopants provide narrow and stable emissions, their inherently low absorption cross sections limit the efficiency. Incorporating them into strongly absorbing metal NCs could achieve energy transfer between the NC and rare-earth dopants,^12^ potentially enabling intriguing upconversion and downconversion properties.

Third, surface ligand functionalization provides an additional handle to expand the NC functionality. For instance, coupling dye molecules to the ligands can greatly enhance energy-transfer efficiency and improve TTA-UC and photocatalysis,^68^ while grafting PEG chains can substantially increase water solubility and biocompatibility.

Overall, continued progress in the synthetic control, compositional tuning, and surface functionalization will further unlock the potential of metal NC materials for next-generation NIR-II photonic technologies with unprecedented capabilities.
